# Causal effect between total cholesterol and HDL cholesterol as risk factors for chronic kidney disease: a mendelian randomization study

**DOI:** 10.1186/s12882-020-02228-3

**Published:** 2021-01-20

**Authors:** Liu Miao, Yan Min, Bin Qi, Chuan-Meng Zhu, Jian-Hong Chen, Guo-Xiong Deng, Yong Wang, Jian-Fei Li, Rong-Shan Li

**Affiliations:** 1 Departments of Cardiology, Liuzhou People’s Hospital, 8 Wenchang Road, Liuzhou, 545006 Guangxi People’s Republic of China; 2 Departments of Nephrology, Liuzhou People’s Hospital, 8 Wenchang Road, Liuzhou, 545006 Guangxi People’s Republic of China; 3 Departments of Cardiology, The First People’s Hospital of Nanning, 89 Qixing Road, Nanning, 530022 Guangxi People’s Republic of China

**Keywords:** Two-sample mendelian randomization, Genome-wide association study, Serum lipid levels, Chronic kidney disease, Causation

## Abstract

**Background:**

While observational studies show an association between serum lipid levels and cardiovascular disease (CVD), intervention studies that examine the preventive effects of serum lipid levels on the development of CKD are lacking.

**Methods:**

To estimate the role of serum lipid levels in the etiology of CKD, we conducted a two-sample mendelian randomization (MR) study on serum lipid levels. Single nucleotide polymorphisms (SNPs), which were significantly associated genome-wide with serum lipid levels from the GLGC and CKDGen consortium genome-wide association study (GWAS), including total cholesterol (TC, *n* = 187,365), triglyceride (TG, *n* = 177,861), HDL cholesterol (HDL-C, n = 187,167), LDL cholesterol (LDL-C, *n* = 173,082), apolipoprotein A1 (ApoA1, *n* = 20,687), apolipoprotein B (ApoB, n = 20,690) and CKD (*n* = 117,165), were used as instrumental variables. None of the lipid-related SNPs was associated with CKD (all *P* > 0.05).

**Results:**

MR analysis genetically predicted the causal effect between TC/HDL-C and CKD. The odds ratio (OR) and 95% confidence interval (CI) of TC within CKD was 0.756 (0.579 to 0.933) (*P* = 0.002), and HDL-C was 0.85 (0.687 to 1.012) (*P* = 0.049). No causal effects between TG, LDL-C- ApoA1, ApoB and CKD were observed. Sensitivity analyses confirmed that TC and HDL-C were significantly associated with CKD.

**Conclusions:**

The findings from this MR study indicate causal effects between TC, HDL-C and CKD. Decreased TC and elevated HDL-C may reduce the incidence of CKD but need to be further confirmed by using a genetic and environmental approach.

**Supplementary Information:**

The online version contains supplementary material available at 10.1186/s12882-020-02228-3.

## Background

Chronic kidney disease (CKD) can be defined as a decreased glomerular filtration rate (GFR) (< 60 mL/min*1.73 m^2^) and affects up to 15% of the population around the world, and the number of cases is increasing [1]. With the continuous deterioration of renal function, most patients have to accept dialysis treatment. There are many reasons for the deterioration of renal function, including dyslipidemia [2]. Recently, many studies have found that most patients with CKD have cardiovascular diseases (CVD) before they develop end-stage renal disease (ESRD), which is related to abnormal lipid metabolism [3]. Although there is a strong association between CKD and serum lipids, this mechanism has not been fully elucidated.

In epidemiological studies, randomized controlled trials (RCTs) are the most powerful way to demonstrate the etiology hypothesis. However, RCTs are more demanding on research design, and the cost of RCTs is higher; therefore, it is difficult to implement RCTs. The application of the Mendelian randomization (MR) method in epidemiological research provides an economical and effective way to solve this problem [4]. The main principle of this method is that different genotypes determine different intermediate phenotypes, and Mendel’s law of independent distribution states that the intermediate genes are randomly assigned to the gametes of the offspring in the process of gamete formation. Therefore, when using the model of “genotype-disease (outcome)” to simulate the model of “intermediate phenotype (exposure)-disease” to conduct causal correlation research, this approach will not be affected by the impact of environmental factors, and the causal sequence is clear [5]. With these situations, MR research is regarded as the best alternative to RCTs by most researchers.

There are several analyses of MR methods, including two-sample Mendelian randomization (TSMR) [6]. Compared with other methods, TSMR has some advantages. First, with the advent of the post-GWASs era, a large number of GWAS data have been published, and the data that we selected are easier to obtain. Second, if we use the association established by observational research to carry out the research phase of two queues, when the sample size of the study is expanded, the efficiency of the test can be improved. In addition, the published GWAS sample size is usually large, and the number of instrument variables (IVs) that can be selected is high, which increases the genetic interpretation of IVs on exposure, can better replace exposure, and is more conducive to the accuracy and reliability of analysis results [7]. In our current study, we assumed that serum lipid levels were associated with the onset of CKD. Next, we used TSMR to estimate the causal effect of serum lipid levels on CKD.

## Methods

### GWAS data

We selected genetic variants associated with serum total cholesterol (TC, *n* = 187,365), triglyceride (TG, *n* = 177,861), HDL cholesterol (HDL-C, n = 187,167), LDL cholesterol (LDL-C, *n* = 173,082), apolipoprotein A1 (ApoA1, *n* = 20,687) and apolipoprotein B (ApoB, n = 20,690) levels and then extracted the corresponding effect sizes for CKD using the largest GWAS summary-level dataset [8, 9]. The data source of this study is based on re-analyzing previously published GWAS; therefore, there is no ethical approval. CKD data (*n* = 117,165) were acquired from the CKDGen consortium (n = 117,165) [10]. CKD was defined as an eGFR based on serum creatinine (eGFRcrea) lower than 60 mL/min/1.73 m^2^. All datasets were obtained from large-scale randomized double-blind trials and population cohort studies based on European descent. Gender, age and body mass index (BMI) should be corrected in regression models of serum lipid GWAS [8, 9], and age and gender were also adjusted in CKDGen. Because of the potential population stratification in our selected datasets, the genome control of each sample is applied to correct for inconsistent test statistics.

### TSMR design

In our current analysis, the IVs provided by genetic variants should contain three assumptions as in our previous research [1, 11] IVs must be strongly associated with exposure [2]; IVs should be without any association with known confounders; and [3] the IVs we selected must be conditionally independent of exposure (serum lipid levels), outcomes (CKD) and confounders. If the IVs contained the second and third assumptions, it may be regarded as independent from pleiotropy.

### Instrument variables

Initially, IVs should be strongly correlated with exposure (serum lipid level). Then, the *P*-value that we selected should be < 5 × 10^− 8^ in the relevant GWAS dataset to ensure the close association between IVs and exposures. After that step, to ensure independence among selected IVs, PLINK 1.90 [12] was performed to calculate the pairwise linkage disequilibrium (LD). If the *r*^*2*^ was greater than 0.001, these SNPs were excluded from our research.

We selected these IVs must be conditionally independent of outcome (CKD), considering the correlated traits of exposures (serum lipid level), and independent of any known confounders. For selected IVs, only exposure factors (serum lipid level) and no other pathways or confounding factors can affect the outcome (CKD). This finding is consistent with the previous two assumptions [13]. First, we made the corresponding effect estimates of these variables on CKD. We should choose the proxy SNPs that are highly correlated (*r*^*2*^ was greater than 0.8) based on the SNP Annotation and Proxy (SNAP) search system for substitution when the selected SNPs cannot be used in CKD [14]. Next, MR-Egger regression was performed to calculate the horizontal pleiotropic [15]. Afterwards, we removed any palindromic SNPs for which the minor allele frequency (MAF) was greater than 0.3 to ensure that the influence of the SNPs on the exposures (serum lipid level) corresponded to the same allele as their influence on CKD [16]. Subsequently, we employed the GWAS Catalog to check for the associations between selected IVs and to adjust for potential confounding. In addition, we calculated the F statistic with a web application (https://sb452.shinyapps.io/overlap/) to examine the association of selected IVs with the exposures [17].

### Pleiotropy assessment

MR-Egger regression was employed to calculate the horizontal pleiotropic pathway between IVs and CKD, independent of serum lipid level [15]. As an effective method to detect bias in publication meta-analysis, MR-Egger regression was derived from Egger regression. The method can be expressed through the equation α_i_ = β_γi_ + β_0_. In this equation, different letters indicate different meanings. α_i_ represented the effect between IVs and CKD; γi was employed to estimate the effect between serum lipid level and IVs; slope β denoted the estimated causal effect of exposure (serum lipid level) on outcome (CKD); and intercept β_0_ represented the estimated average value of horizontal pleiotropic. When the *P*-value of the intercept was greater than 0.05, no horizontal pleiotropy could be found. In addition, the slope can also be defined as the estimated pleiotropy-corrected causal effect. However, if the SNPs we selected in this analysis do not account for most of the differences in exposure, then there is a lack of evaluation of this estimate [15].

### TSMR analysis

In our current study, inverse variance weighted (IVW) was used as the key method to calculate the causal effect between serum lipid level and CKD for TSMR analysis [18]. The causal effect β was estimated and shown as w_i_ (α_i_ /γ_i_). In this equation, i refers to the IVs, α_i_ represents the association effect of IVs on CKD, γ_i_ defines the association effect of IVs on serum lipid level, and w_i_ represents the weights of the causal effect of serum lipid level on CKD.

### Sensitivity analysis

We employed various methods to calculate follow-up sensitivity, including maximum likelihood, MR Egg, weight median, penalized weight median, simple mode, weight mode and robust adjusted profile score (RAPS) [19]. Compared with IVW, these methods have greater robustness to individual genetics with strongly outlying causal estimates and would generate a consistent estimate of the causal effect when valid IVs exceed 50% [20]. Then, leave-one-out sensitivity analysis was performed to screen out whether the correlation was out of relationship to be affected by a single SNP. Subsequently, we employed TSMR analysis again, leaving out each SNP, in turn, and the overall analysis including all SNPs was shown for comparison [21]. All of the analysis was implemented by the “TwoSampleMR” package in the R software environment.

## Results

### IV selection and validation

Seven independent SNPs (*P* < 5 × 10 ^− 8^, *r* ^*2*^ < 0.001) were associated with TC, thirteen independent SNPs were associated with TG, four SNPs were associated with HDL-C, four SNPs were associated with LDL-C, seven SNPs were associated with ApoA1 and nine SNPs were associated with ApoB by independent and LD analyses (Supplementary Table 1).

Then, we employed the intercept term to calculate the exposures from MR-Egger regression. Table 1 shows the MR-Egger regression intercepts and indicates that no horizontal pleiotropy exists in the current TSMR analysis. Table 2 identified the heterogeneity tests and found that all the *P*-values were greater than 0.05.

**Table 1:** Mendelian randomization (MR)-Egger regression intercepts

Exposure	Outcome	Intercepts (95% CI)	*P*-val
TC	CKD	0.015 (−0.113, 0.066)	0.573
TG	CKD	−0.008 (− 0.047, 0.019)	0.553
LDL-C	CKD	−0.032 (− 0.105, 0.037)	0.461
HDL-C	CKD	−0.041 (− 0.150, 0.056)	0.492
ApoA1	CKD	0.009 (−0.088, 0.049)	0.682
ApoB	CKD	0.008 (−0.078, 0.036)	0.733

*CKD* Chronic kidney disease, *TC* Total cholesterol, *TG* Triglyceride, *HDL-C* High-density lipoprotein cholesterol, *LDL-C* Low-density lipoprotein cholesterol, *Apo* Apolipoprotein, *CI* Confidence interval, *MR* Mendelian randomization; The significant result (*P* > 0.05) indicates that the y-intercept of the MR-Egger regression line is not significantly different from zero and thus no pleiotropy exists

**Table 2:** Heterogeneity tests

Type	Method	Q	Q_df	Q_pval
TC	MR Egger	5.410	5	0.367
	Inverse variance weighted	5.803	6	0.445
TG	MR Egger	16.062	11	0.138
	Inverse variance weighted	16.609	12	0.164
LDL-C	MR Egger	1.462	2	0.481
	Inverse variance weighted	2.281	3	0.516
HDL-C	MR Egger	0.301	2	0.859
	Inverse variance weighted	0.997	3	0.801
ApoA1	MR Egger	2.132	5	0.831
	Inverse variance weighted	2.320	6	0.887
ApoB	MR Egger	13.726	7	0.056
	Inverse variance weighted	13.973	8	0.082

*TC* Total cholesterol, *TG* Triglyceride, *HDL-C* High-density lipoprotein cholesterol; *LDL-C* Low-density lipoprotein cholesterol, *Apo* Apolipoprotein, *CI* Confidence interval, *MR* Mendelian randomization; The significant result (*P* > 0.05) indicates no heterogeneity exists

Subsequently, we performed F statistics to identify the strength of the relationship between IVs and exposures. If F > 10, it should be considered to be strong enough to mitigate against any bias of the causal IV estimate. The F statistics for our selected IVs were 107,061.14 for TC, 54722.15 for TG, 173077 for HDL-C, 187162 for LDL-C, 11816.57 for ApoA1 and 9191.11 for ApoB. All the F statistics values were greater than 10, which indicated high strength to mitigate against any bias of the causal IV estimate.

### TSMR and sensitivity analysis

**Fig. 1:**
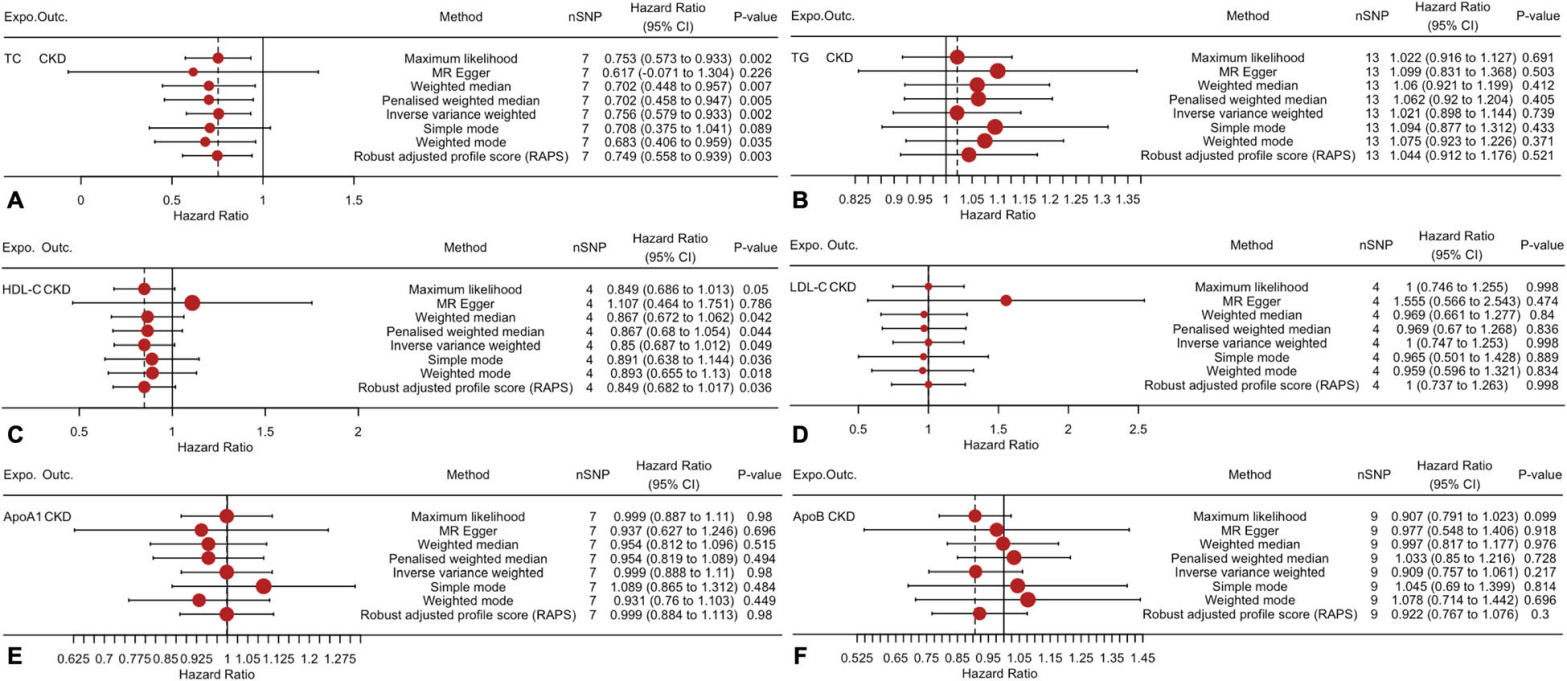
Two-sample mendelian randomization of serum lipid level and the risk of CKD. **a** TC; **b** TG; **c** HDL-C; **d** LDL-C e ApoA1; **f** ApoB. *Expo.,* exposure; *Outc.*, outcome. *CKD*: Chronic kidney disease; *TC*, Total cholesterol; *TG*, Triglyceride; *HDL-C*, High-density lipoprotein cholesterol; *LDL-C*, Low-density lipoprotein cholesterol; *Apo*, Apolipoprotein; *CI*: Confidence interval; *MR*: Mendelian randomization

The TSMR analysis results are shown in Fig. 1. The odds ratio (OR) and 95% confidence interval (CI) of TC within CKD was 0.756 (0.579 to 0.933) (*P* = 0.002), TG was 1.021 (0.898 to 1.144) (*P* = 0.739), HDL-C was 0.85 (0.687 to 1.012) (*P* = 0.049), LDL-C was 1 (0.747 to 1.253) (*P* = 0.998), ApoA1 was 0.999 (0.888 to 1.11) (*P* = 0.98) and ApoB was 0.909 (0.757 to 1.061) (*P* = 0.217). These results suggested that this method genetically predicted the causal effect between TC/HDL-C and CKD.

**Fig. 2:**
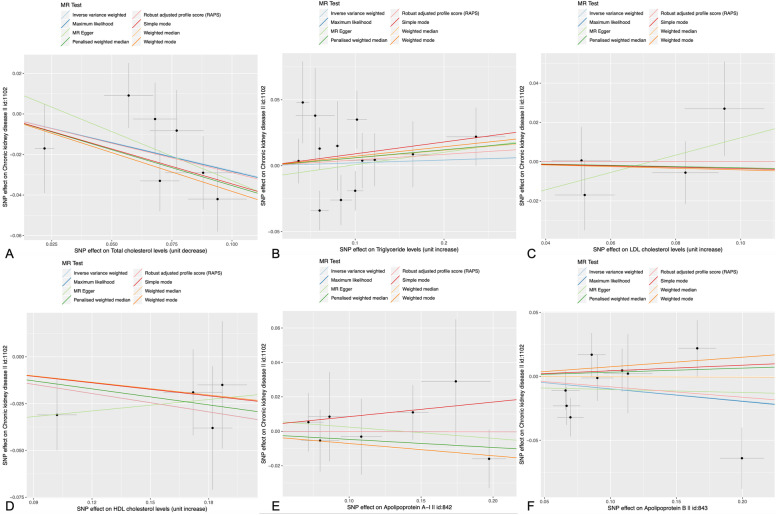
Scatter plots of the estimated SNP effects on serum lipid level (x-axis) plotted against the estimated SNPs effects on the CKD (y-axis). **a** TC; **b** TG; **c** HDL-C; **d** LDL-C (**e**) ApoA1; **f** ApoB. The slope of the line corresponds to a causal estimate using a different method

**Fig. 3:**
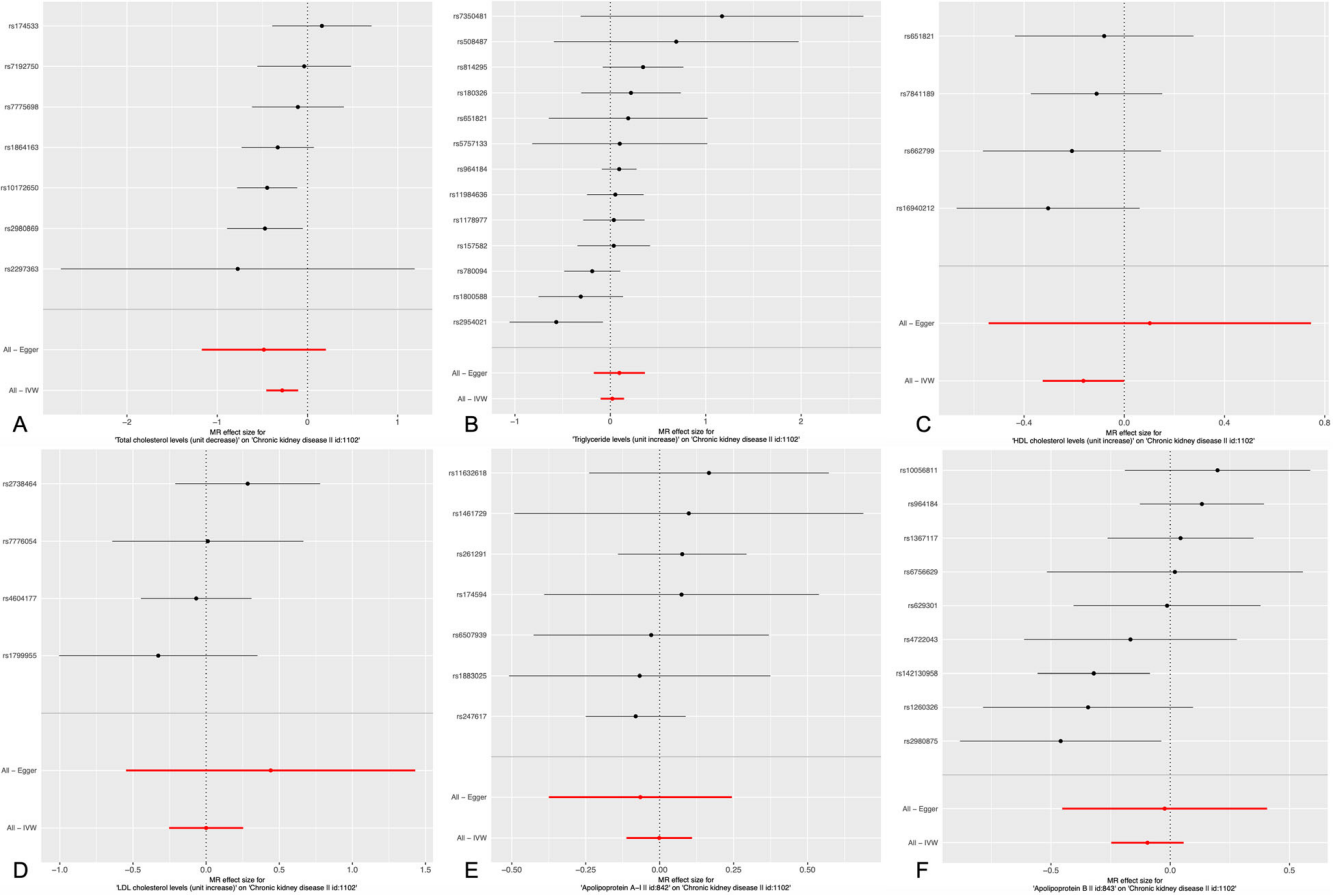
Results of the single and multi SNP analyses for the SNP effect of serum lipid level on CKD. **a** TC; **b** TG; **c** HDL-C; **d** LDL-C (**e**) ApoA1; **f** ApoB. The forest map, where each black dot represented a single SNP as IV, showed the logarithm of the odds the ratio 95% (OR) confidence per standard deviation under the influence of serum lipid level; the red dot showed the use of IVW results for all SNPs; the horizontal line indicated the 95% confidence interval

**Fig. 4:**
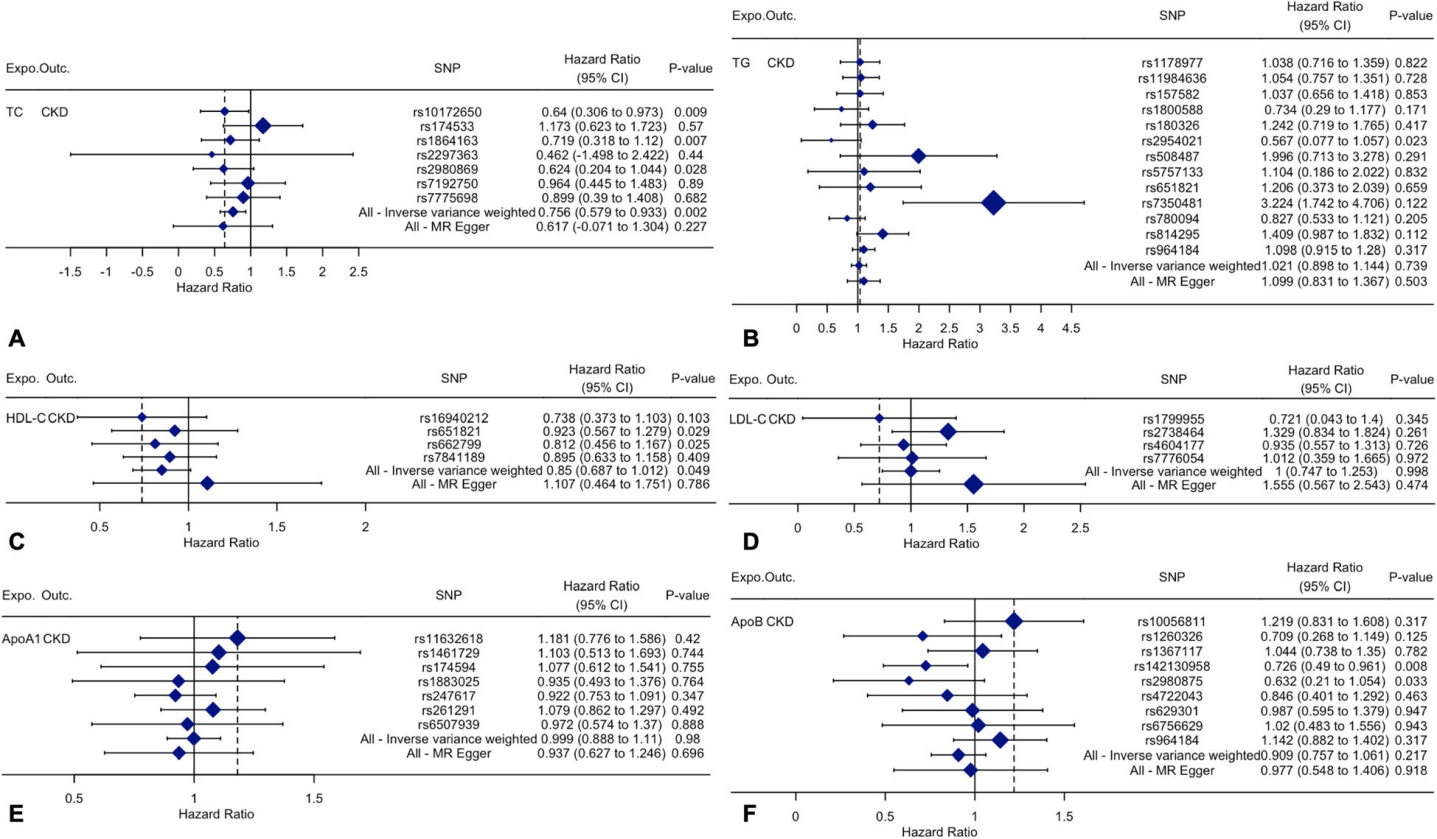
The details of SNP analyses for the SNP effect of serum lipid level on CKD**. a** TC; **b** TG; **c** HDL-C; **d** LDL-C (**e**) ApoA1; **f** ApoB. *Expo.,* exposure; *Outc.*, outcome. *CKD*: Chronic kidney disease; *TC*, Total cholesterol; *TG*, Triglyceride; *HDL-C*, High-density lipoprotein cholesterol; *LDL-C*, Low-density lipoprotein cholesterol; *Apo*, Apolipoprotein; *CI*: Confidence interval; *MR*: Mendelian randomization

**Fig. 5:**
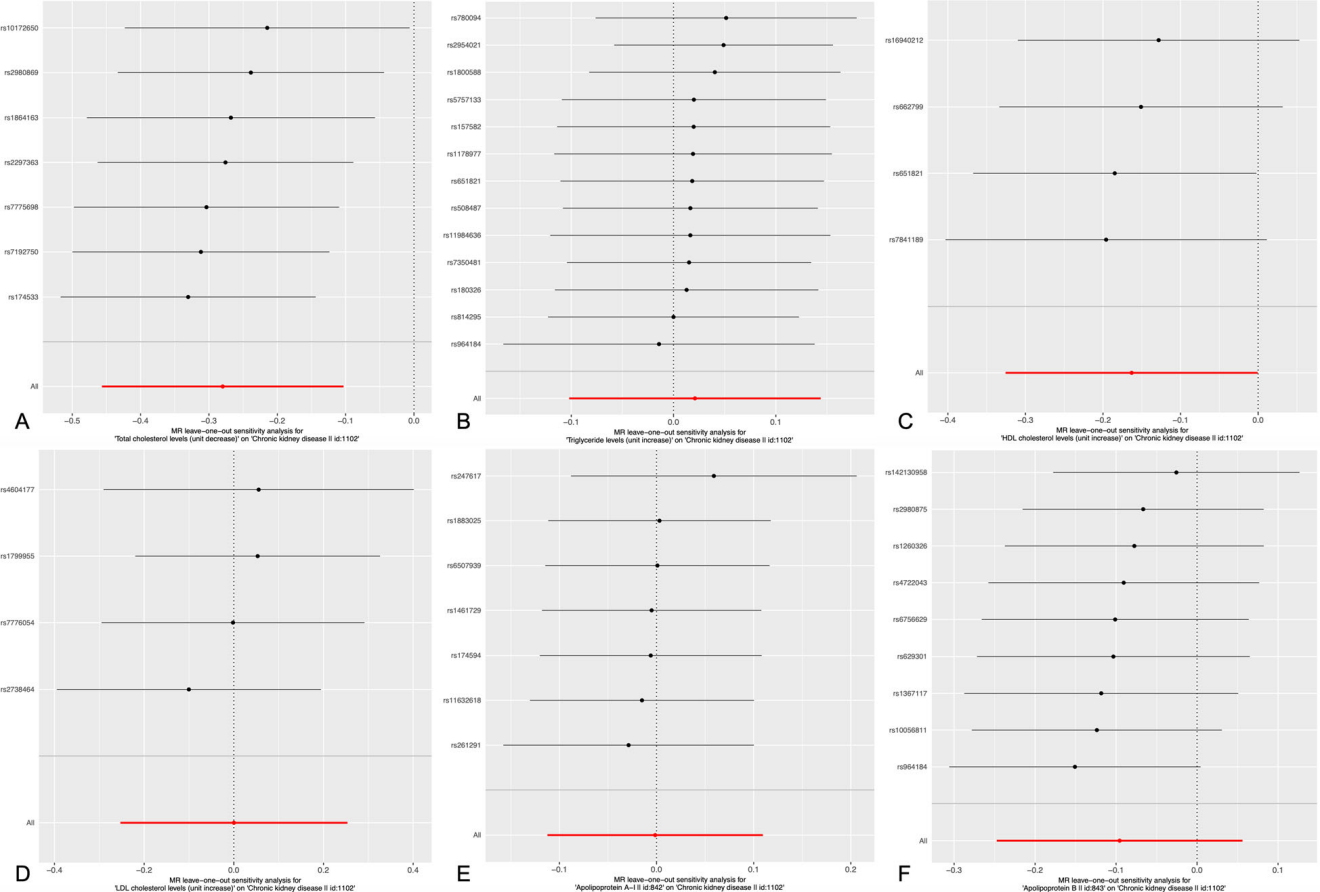
Sensitivity analyses using the leave-one-out approach on the association of serum lipid level on CKD. **a** TC; **b** TG; **c** HDL-C; **d** LDL-C (**e**) ApoA1; **f** ApoB. Each black dot represents an IVW method for estimating causal the effect of the line serum lipid level on the CKD does not exclude a case where a particular SNP caused a significant change in the overall results

**Fig. 6:**
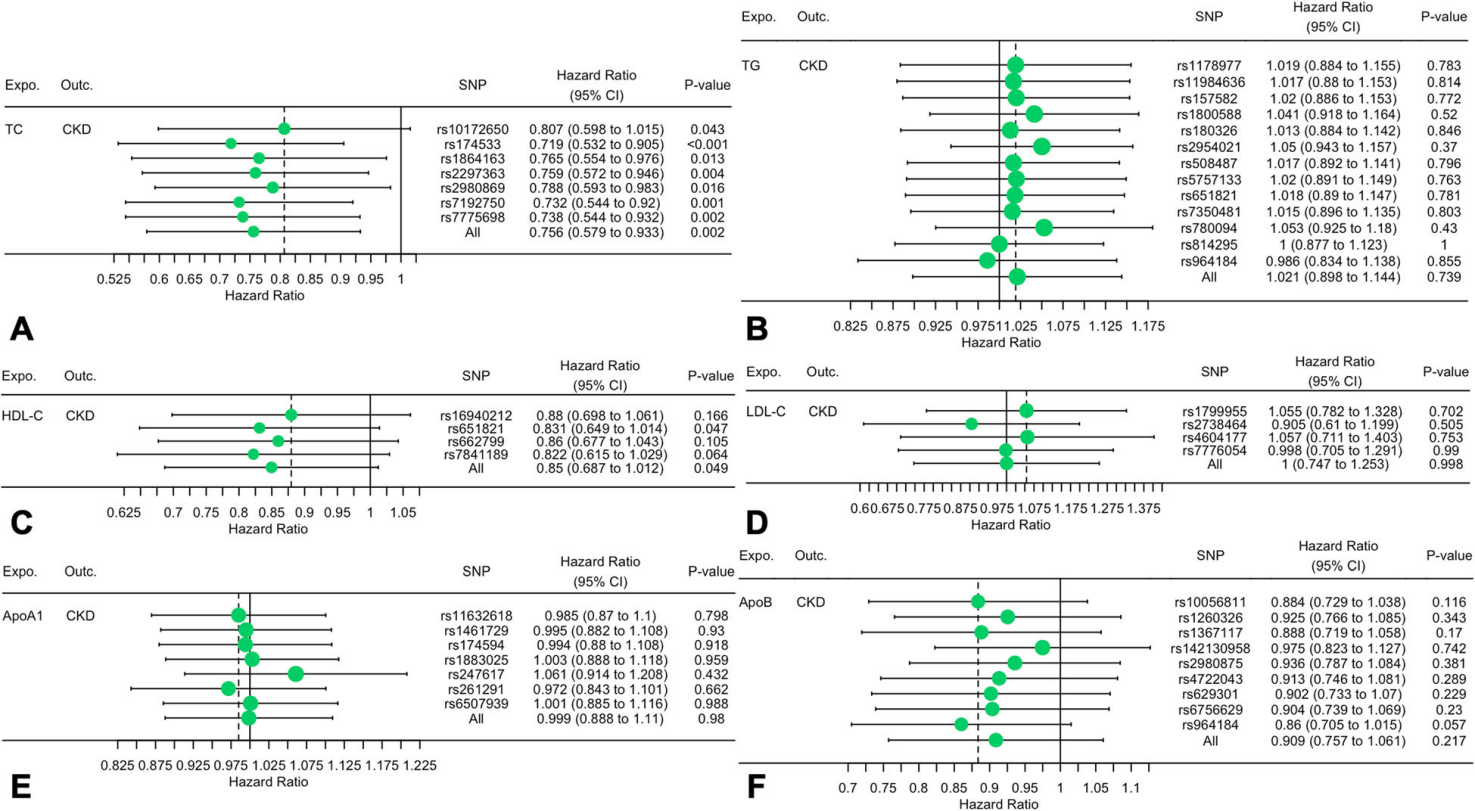
The details of Sensitivity analyses for the leave-one-out approach on the association of serum lipid level on CKD (**a**) TC; **b** TG; **c** HDL-C; **d** LDL-C (**e**) ApoA1; **f** ApoB. *Expo.,* exposure; *Outc.*, outcome. *CKD*: Chronic kidney disease; *TC*, Total cholesterol; *TG*, Triglyceride; *HDL-C*, High-density lipoprotein cholesterol; *LDL-C*, Low-density lipoprotein cholesterol; *Apo*, Apolipoprotein; *CI*: Confidence interval; *MR*: Mendelian randomization

Figure 2 indicates the association between serum lipid levels and CKD and shows that there is a positive correlation between a decrease in TC and an increase in HDL-C and the incidence of CKD. The overall estimates, as calculated by IVW or MR-Egg, also reveal causal effects between serum lipid levels and CKD. (Figs. 3 and 4). Sensitivity analyses using the leave-one-out approach confirmed the causal effect (Figs. 5 and 6).

## Discussion

There is a consensus in previous studies that dyslipidemia is an independent risk factor for CVD. With further research, the contribution of decreased HDL, increased LDL and cholesterol to CVD is more obvious [22]. At present, studies have only found that dyslipidemia is closely related to CKD, but whether serum lipids can directly lead to CKD has not been determined.

Patients with CKD have dyslipidemia even at early stages of renal disease and dyslipidemia tends to progress with deterioration of kidney function. The dyslipidemia in CKD is largely due to increased triglyceride levels, decreased HDL-C and varying levels of LDL-C. There are many national guidelines for treatment of dyslipidemia in the general population as well as those with CKD and collectively the guidelines advocate for the use of statins as first line therapy in patients with ASCVD or at high risk for ASCVD. The guidelines that included CKD as a specific at-risk population support the use of statins to reduce ASCVD risk in those with pre-end stage CKD and in those post renal transplant [23]. As early as the early 1990s, there was a case report that there was a relationship between hyperlipidemia and CKD. After ten years of follow-up, it was found that the incidence of albuminuria in hypercholesterolemia, hypertriglyceridemia and low-HDL-C was higher than that in the normal group, regardless of gender [24]. Another large, community-based cross-sectional study in Japan also found that hyperlipoproteinemia is closely related to the decline of eGFR [25]. Mendy et al. also found that the correlation between hypercholesterolemia and CKD was not related to race or skin color [26]. With further research, evidence obtained in mouse models has emerged suggesting that renal damage is caused by serum lipids. The proximal renal tubules are the most easily damaged sites of dyslipidemia. Serum lipid accumulation will lead to damage to renal tubules and aggravation of interstitial fibrosis, which will contribute to a decrease in eGFR [27, 28].

In epidemiology, RCT is the most authoritative method to prove the etiology hypothesis. However, RCTs often require a large sample size, a more rigorous experimental design procedure, a longer follow-up time and a higher cost, which inhibits researchers from conducting RCTs, thereby limiting the verification of many hypotheses. In recent years, with the continuous updating of research methods, MR has been recognized as the best alternative to RCT [29]. One of the important processes of MR is to select IVs, and single nucleotide polymorphism is one of the most commonly used IVs. In addition, there is abundant GWAS research related to serum lipids, and as the sample size is large enough, it has considerable credibility for the inference of MR.

In fact, some risk factors related to CKD have been found by using MR. Jordan et al. found that using genetics does not support a causal effect of serum urate level on eGFR level or CKD risk, and reducing SU levels is unlikely to reduce the risk of CKD development [30]. This finding is not in keeping with our traditional understanding. Del Greco et al. showed a 1.3% increase in eGFR per standard deviation increase in iron (95% confidence interval 0.4–2.1%, *P* = 0.004), which suggests a protective effect of iron on kidney function in the general population [31]. There are also some studies on the relationship between serum lipids and CKD, but there are some differences with our research focus. Lanktree et al. found that higher HDL cholesterol concentration was causally associated with better kidney function, and there was no association between genetically altered LDL cholesterol or triglyceride concentration and kidney function [32]. But serum HDL and CKD mortality show a U-shaped curve, with elevated HDL reducing the mortality rate of CKD within a certain range, while persistently elevated HDL significantly increases the risk of death from CKD [33]. In the conclusion of HDL-C, it is the same as our current research. Compared with the studies by this researcher, we used IVW to make a direct inference and used a variety of methods to verify this conclusion. At the same time, we made a judgment on the level of the horizontal pleiotropic pathway and sensitivity, avoiding the interference of false negatives with the conclusion. Liu et al. [34] also found that HDL-C is related to the pathogenesis of CKD, while TC, TG and LDL-C are not. Compared with the studies conducted by these researchers, there are some differences in the selected GWAS results in our current study, and we paid more attention to the correlation between the change range and trend of serum lipids and the incidence of CKD to guide the clinical diagnosis and treatment.

There were several limitations in our studies. First, as we can only download the data collected from the website for analysis, in this case, we cannot obtain the clinical result value of each individual in the original data; therefore, we cannot perform further analysis according to the subtype of CKD. Second, different standards of quality control in individual-level GWAS may affect our results. Third, these results only consider the causal relationship between serum lipid levels and CKD. In fact, the pathogenesis of CKD is a highly complex process, and other pathogenic factors need to be evaluated. The results of this study provide a new vision for the clinical understanding of the relationship between serum lipid levels and CKD and provide a theoretical basis for clinical decision-making.

## Conclusion

By performing TSMR analysis, we identified that serum TC and HDL-C are causally associated with CKD risk. Decreased TC and elevated HDL-C may reduce the incidence of CKD. However, additional human and animal studies are still needed to further confirm these results by using a genetic and environmental approach.

## Supplementary Information

**Additional file 1: Supplemetary Table 1.** The details of SNP IVs.

## Data Availability

The datasets generated and/or analysed during the current study are publicly available.
